# Mechanochemists Want to Shake up Industrial Chemistry

**DOI:** 10.1021/acscentsci.2c01273

**Published:** 2022-11-02

**Authors:** Fernando Gomollón-Bel

Grinding stuff is probably one of the oldest ways to run a chemical reaction.

For example, in the fourth century BCE, people extracted mercury from cinnabar by grinding the mineral with vinegar in copper bowls. Later, as the chemical industry developed in the 19th century, the German chemistry company, BASF, used giant mills called mühlenbetriebe to grind materials into synthetic organic dyes. Factory workers would load a ball mill’s large cylindrical drum with starting material and metal balls. The balls would grind the materials as the drum spun. One of the dyes made in these mills was Heliogen blue, an intense sapphire dye made by crumbling copper chloride and phthalonitrile.

“It is a really old example of a mechanochemical reaction, run without a solvent in a ball mill kiln,” Martin Viertelhaus, a principal scientist at BASF, said at the “Mechanochemistry Meets Industry” online workshop in February 2021.

In recent decades, this approach, called mechanochemistry, has made a resurgence in academic laboratories. Because this unusual chemical method relies simply on grinding compounds, it avoids high temperatures and solvents, and offers more environmentally friendly alternatives to traditional synthetic routes. Some researchers have also reported that mechanochemistry can facilitate transformations not available with conventional methods. Mechanochemistry “achieves fairly new, incredible things,” says John Warner, a chemist at Zymergen and one of the coauthors of the *12 Principles of Green Chemistry*.

But the technology developed in academic labs faces a scale-up hurdle before it can reach industry. Experts think that industrial implementation of new mechanochemical methods will require investment and innovation. Around the world, chemists are building bridges between academia and industry to speed up mechanochemistry’s adoption.

Industry has been interested in mechanochemistry in part because of its greenness. María Elena Rivas Velazco, a principal scientist at Johnson Matthey, and her team investigate mechanochemical syntheses of mixed oxide materials for applications in catalysis, alloys, and batteries. She says they have explored mechanochemistry because it offers “production routes with smaller environmental footprints, [which] use fewer hazardous reactants and result in reduced levels of waste.” Mechanochemical processes are usually more sustainable because they avoid using excess reagents, solvents, and tedious purification methods.

This approach also achieves good atom economy, says Evelina Colacino, a researcher at the University of Montpellier. Atom economy measures how many of the atoms in the starting material end up in the product. More atoms that end up in the product means less waste and a more sustainable process. For example, mechanochemistry enables condensation reactions between amines and carbonyls without using a base, which is generally needed to spark the transformation in solution. The base doesn’t get incorporated into the resulting amide, so it decreases atom economy.

In addition to mechanochemistry’s green advantages, the approach can also unlock transformations that are unattainable by other means. “It opens the door to a new chemical space,” Colacino says. Mechanochemistry can open these doors because mechanical forces instead of heat, light, or electricity drive reactions.

Recent studies suggest that the kinetics and thermodynamics of a reaction inside a ball mill don’t follow well-known rules of organic chemistry, and sometimes these deviations can lead to unexpected products. Mechanochemistry, for example, can functionalize fullerenes via routes that don’t work in solution. Other aspects of reactions, like where on a molecule a modification occurs, also change under mechanical pressure. This control of site selectivity is especially important for the development of new drugs. “Mechanochemistry creates a lot of opportunities for the pharmaceutical industry, potentially leading to new active ingredients,” Colacino says.

Deborah E. Crawford, a researcher at the University of Bradford, found a family of copper complexes whose structures—and thus anticancer efficacies—depend on how they’re made. “Copper metallodrugs form square-planar structures in solution, while ball mills yield octahedral complexes that are more active against cancer,” she says.

Researchers at Johnson Matthey are collaborating with Crawford and Colacino to synthesize other new, pharmaceutically active compounds using mechanochemistry. “This is still exploratory,” Velazco says. They are being cautious because drug molecules’ bioactivity in the body could change when the molecules are prepared with solvent-free techniques. But the scientists are experimenting with the approach. “It will likely take time to deploy this industrially,” Crawford says.

To help speed up mechanochemistry’s move from the research lab to industry, chemists are seeking ways to make the chemistry even more attractive to chemical companies. For example, Colacino aims to bridge the gap between academia and industry by coordinating a European project called Mechanochemistry for Sustainable Industry. It is funded under COST (European Cooperation in Science and Technology), a program that promotes the creation of an interdisciplinary research network. “We want to establish a collaborative community to share knowledge in this up-and-coming field and ensure the technology is profitable,” she says. Since the project’s creation in 2019, it has doubled the participant countries and institutions, to more than 400 people from 38 countries worldwide.

“We [have] observed a growing interest from scientists in the private sector,” Colacino adds. Companies such as Syngenta, Solvay, Teva Pharmaceuticals, and Johnson Matthey have participated in the organization’s events. Furthermore, this project catalyzed the creation of a European consortium dubbed Impactive, led by the University of Montpellier and including companies like Novartis. The consortium, which will be funded with almost $8.5 million (€8 million) from the European Commission’s Horizon Europe program, will launch later this year and will develop mechanochemical processes to sustainably produce pharmaceutical ingredients.

In the US in 2020, the National Science Foundation awarded a $1.8 million grant to start the NSF Center for the Mechanical Control of Chemistry (CMCC), led by Texas A&M University chemist James D. Batteas. “Our aim is to understand the fundamentals of mechanochemistry to better predict the outcome of reactions and enable better design of reactors,” Batteas says. In one early signal of the venture’s success, the CMCC has patented a new type of mechanochemical reactor that incorporates a magnetic force sensor to measure and understand the collisions between reagents. Eventually, the CMCC hopes to do more in translational research and strengthen ties with the chemical industry.

To bring new mechanochemical methods to industry, however, chemists will have to overcome some hurdles. Companies will need to modify established chemical processes and replace their current equipment at plants with ball mills and other mechanochemical equipment. Although some companies already use industrial-scale mills and extruders, most of these changes require investments of money and time, especially because new methods to produce pharmaceuticals will need to get approval by government regulators.

But one of the biggest hurdles blocking the advance of mechanochemistry is the lack of demonstrations of the chemistry on large scales, says Valerio Isoni, an expert in process development and team leader at the Institute of Sustainability for Chemicals, Energy, and Environment (ISCE^2^) in Singapore.

Isoni thinks industry could repurpose and retrofit existing equipment to meet some of these large-scale needs. One example is the extrusion equipment used in the production of bread dough and the fabrication of plastics from polymer pellets. This type of gear uses big screws to blend ingredients. For years, academic chemists have tried to adapt extruder screws to mechanochemical reactions. Instead of a ball mill grinding materials, two screws in an extruder would mash reagents together.

**Figure:**
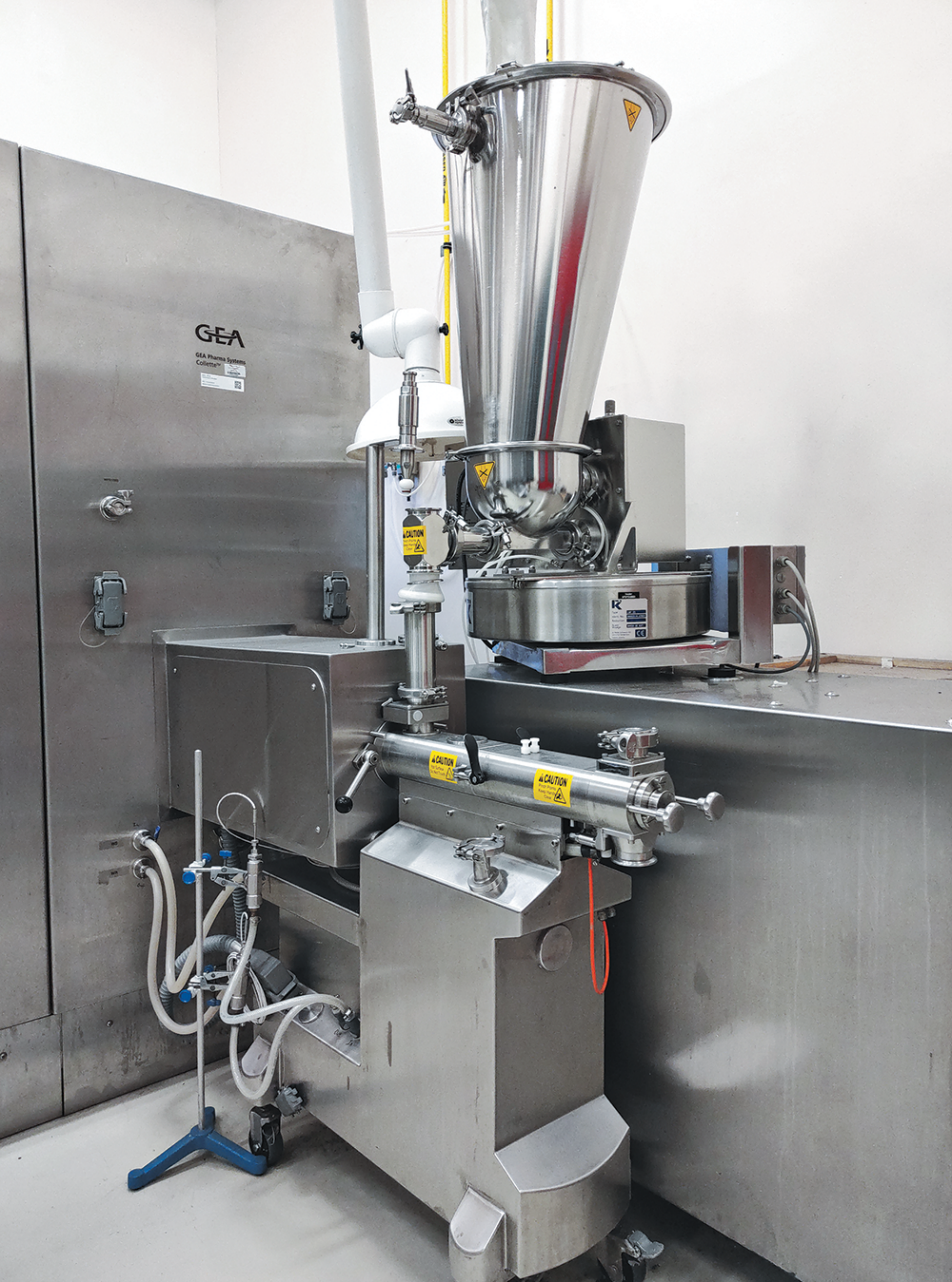
Valerio Isoni’s lab in Singapore uses a twin screw granulator for mechanochemistry. Credit: Courtesy of Valerio Isoni

Ball mills operate in batch, meaning they need loading and unloading, like traditional chemical reactors. But extruders operate continuously and allow for more controlled conditions throughout the process.

As a postdoc at Queen’s University Belfast, Crawford studied how to adapt extruder screws to mechanochemistry. Her team developed devices that push extrusion backward, allowing starting materials to mash together for longer when reactions need extra time. The researchers also used Raman spectroscopy to monitor chemical changes within an extruder in real time.

Eventually, these efforts bore fruit. In 2021, the team reported a solvent-free, continuous synthesis of perylene dyes at a rate of 1.5 kg per day, which is up to twice the rate of solvent-based batch methods. Also, in collaboration with Colacino, the team used extrusion to prepare value-added pharmaceuticals, including the antibiotic nitrofurantoin and the muscle relaxant dantrolene, in the lab. This procedure produced the molecules at about 0.3 kg per day.

Some companies are starting to try mechanochemistry at industrial scales with pilot plants. For example, MOF Technologies, based in Belfast, Northern Ireland, applies extrusion methods to manufacture metal–organic frameworks. The company can produce about 15 kg of materials per hour, which is enough to supply customers and research partners but still needs to be scaled up further for other industrial applications. The company has several patents for both ball milling and extrusion processes, Chief Technology Officer José Casabán says. The company uses mechanochemistry to make metal–organic frameworks for a wide variety of applications, including gas adsorption, filtration, energy, and drug delivery. Casabán says the company has collaborated with other firms, including General Motors, IBM, Cemex, and ArcelorMittal.

**Figure:**
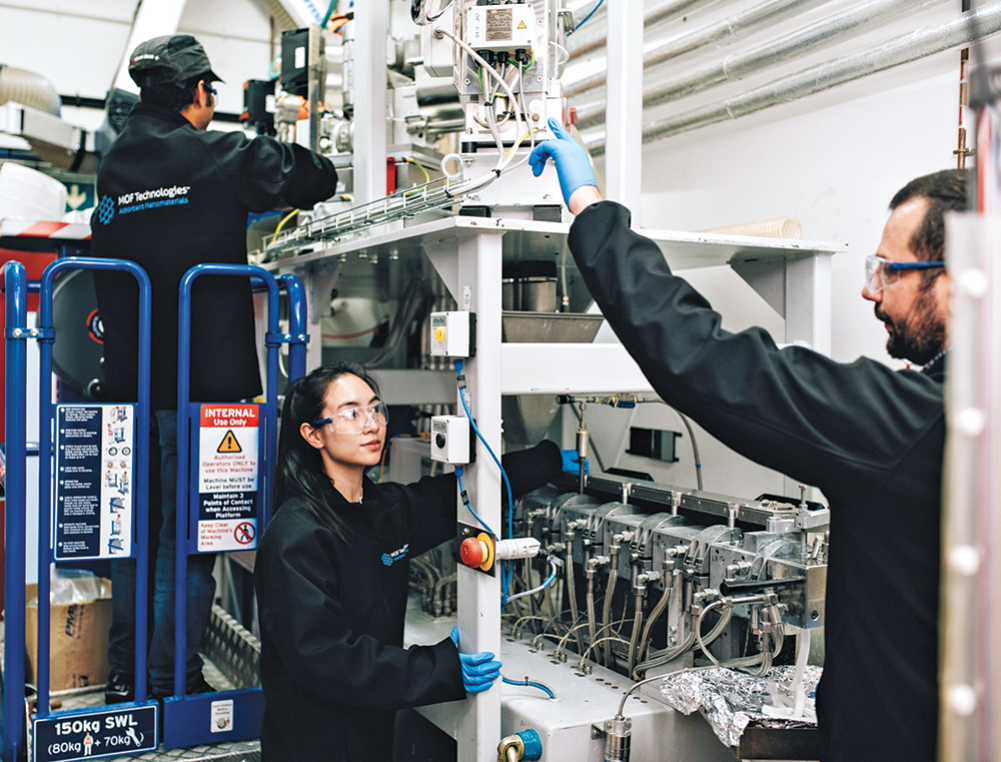
Researchers at MOF Technologies work with extruders to use mechanochemistry to develop new materials. Credit: MOF Technologies

Meanwhile, at ISCE^2^, scientists have explored mechanochemical alternatives to traditional chemical reactions for making drug molecules. The researchers have collaborated with several companies, including Pfizer, GSK, and Merck & Co. These types of projects are the first steps to bringing this chemistry to industry. “We want to show [that] mechanochemistry is ready for scale-up,” Isoni says.

*Fernando Gomollón-Bel is a freelance contributor to**Chemical & Engineering News**, the independent news outlet of the American Chemical Society. A version of this story appeared in C&EN.*

